# Association between positive youth development and family functioning in the Chinese context: a four-wave longitudinal survey in mainland China

**DOI:** 10.3389/fpsyg.2024.1495939

**Published:** 2024-12-11

**Authors:** Xiang Li, Yi-Ting Tang, Daniel T. L. Shek

**Affiliations:** Department of Applied Social Sciences, The Hong Kong Polytechnic University, Kowloon, Hong Kong SAR, China

**Keywords:** preadolescents, adolescents, positive youth development, family functioning, latent growth curve modeling

## Abstract

**Introduction:**

There is a notable scarcity of research examining the developmental trajectories of positive youth development (PYD) attributes among Chinese preadolescents and adolescents and the predictive effect of family functioning on these trajectories over time.

**Methods:**

Using four waves of data, this longitudinal study investigated preadolescents and adolescents in China in January 2020 (T1), June 2020 (T2), June 2021 (T3), and June 2022 (T4). The study utilized the 90-item “Chinese Positive Youth Development Scale” and the 33-item “Chinese Family Assessment Instrument” to assess PYD and family functioning, respectively. The final matched sample comprised 2,652 Chinese students from grades 4 to 8, with an average age of 10.9 years (SD = 1.32; range 9–15) at the first wave of the survey, and with 51.1% male (*n* = 1,354).

**Results:**

The Latent Growth Curve Modeling (LGCM) revealed that the PYD developmental trajectories of preadolescents and adolescents followed a quadratic U-shaped curve, characterized by an initial decline from T1 to T3, followed by a rebound from T3 to T4. Although the time-invariant covariate LGCM indicated no significant gender difference in the initial level of PYD, girls exhibited a slower decline rate and a faster growth rate in PYD over time than did boys. The parallel LGCM demonstrated that initial levels of family functioning significantly and positively predicted both the initial level and the rate of change in PYD over time.

**Discussion:**

This study highlights the critical importance of considering the direct and sustained impact of family functioning within Chinese contexts on positive developmental outcomes among Chinese preadolescents and adolescents. This study also suggests that when designing and formulating specific programs or interventions, it is essential to consider gender differences in the development of competencies to ensure the optimal development of young individuals of different genders.

## 1 Introduction

A core principle underlying the “Positive Youth Development (PYD)” approach is that every child possesses natural talents, strengths and interests (Damon, 2004). The theoretical models of PYD posit that when young individuals interact adaptively with their multi-level environments, positive developmental trajectories of improvement are more likely to occur (Lerner et al., 2003; Lerner, 2004). In particular, PYD emphasizes nurturing the competencies of young individuals, along with providing them with comprehensive support systems to enable them to unleash their full potential, achieve positive and healthy development, and successfully transition into adulthood.

As the basic and most crucial developmental context for children and adolescents, the family directly and profoundly influences their developmental outcomes. The family provides essential assets and a support system necessary for children’s positive development and can initiate PYD trajectories. In this context, the family environment such as family interactions not only directly impact children’s developmental outcomes but may also have long-lasting effects on their future well-being. In Chinese communities, the highly child-centered nature of Chinese families may be a double-edged sword, offering a supportive and protective environment while also potentially leading to excessive parental control. Therefore, it is crucial to explicitly examine how family functioning influences developmental outcomes among Chinese young individuals over time.

### 1.1 Positive youth development—an alternative vision of youth development

A new perspective on viewing young individuals has emerged since the early 1990s, which considers every child and adolescent as a social resource with untapped potential for positive growth (Lerner et al., 2005). This developmental view takes the concepts of plasticity and resilience in human development as its foundation and focuses on the strengths of young individuals (Lerner et al., 2015), and highlights the malleability, diversity, and self-determination inherent in young individuals’ developmental processes (Steinberg and Lerner, 2004). According to Hamilton’s (1999) discussion, the term “Positive Youth Development (PYD)” can be interpreted from at least three aspects. Firstly, it is a developmental process. Secondly, PYD represents an ideology and serves as a guiding principle for designing and implementing youth programs. Thirdly, PYD exemplifies those youth programs that emphasize nurturing the abilities of young individuals and promoting their positive and harmonious development.

There are different approaches in the field of PYD, with the Five Cs Model (Lerner, 2004; Lerner et al., 2005) and the 15 PYD constructs (Catalano et al., 2004) being the most widely utilized and improved upon. The Five Cs Model conceptualizes PYD through five key components: “competence,” “confidence,” “character,” “connection,” and “caring” (Lerner et al., 2005) These five Cs are considered essential developmental attributes that young individuals need to become contributing members of society. Catalano et al. (2004), based on their review of the effective programs in the United States, identified 15 developmental constructs as indicators of PYD, such as cognitive, emotional, behavioral, social and moral competence. Scholars operationalize and define PYD from various perspectives, however, there is a consensus on two common emphases across different PYD models. Plasticity, the capacity to adapt to different environments, is regarded as an inherent potential in human development and is commonly emphasized within various conceptual frameworks of PYD. Taking into account the entire lifespan, change is always present, and the direction of change toward improvement (a positive change) or deterioration (a negative change) can become a characteristic of an individual’s developmental trajectory. The complexity and diversity of the “relational developmental system” provide possibilities for identifying pathways of positive change for individuals, thereby promoting or sustaining positive development (Lerner et al., 2015). Hence, the second common emphasis in various PYD models is the relationship between individuals and their contexts. Within this “relational developmental system,” adaptive developmental regulations occur when individuals and their developmental contexts are mutually beneficial (Brandtstädter, 2007), leading to positive and healthy development. Therefore, the relative plasticity of young individuals is seen as an instantiation of the bidirectional exchanges between them and their developmental contexts (Lerner et al., 2005). When there is consistency and mutual benefit between the ecological assets that promote positive development and individual strengths, which is realized through the adaptive individual-contextual developmental regulations, the important qualities for positive development, such as the 5 Cs (Lerner et al., 2005) and 15 PYD attributes (Catalano et al., 2004), can continue to develop in the individual’s developmental trajectory, thereby leading to the realization of idealized adulthood.

### 1.2 Developmental trajectory of PYD during preadolescence and adolescence

There is currently no universally accepted international definition for the youth age group. The World Health Organization (WHO, 2021) defines “adolescents” as individuals aged between 10 and 19 years old, and “youth” as those in the 15–24 years age group. The term “young people” typically encompasses both of these groups. Additionally, some scholars define the preadolescent age range as 9–12 years old (Buhrmester, 1990; Verburgh et al., 2014) or 10–12 years old (McMakin and Alfano, 2015), typically from Grade 4 to Grade 6, thereby distinguishing this subset of young individuals more precisely from children and adolescents.

Existing empirical research on the developmental trajectories of PYD in young people has primarily focused on the longitudinal investigation, such as the “4-H Study of Positive Youth Development” conducted in the United States since 2002. The study was designed to track the second decade of the youth’s lives to capture their developmental trajectories and examine the individual and ecological factors that promote health and positive growth (Lerner et al., 2005). Multiple findings were observed in this longitudinal study. Based on data from the first three waves, Phelps et al. (2007) found an overall nonlinear decline in PYD developmental trajectories from Grades 5 to 7, with a larger decrease from fifth to sixth grade. Furthermore, it was observed that girls are more likely to be on “positive” developmental trajectories compared to boys. Zimmerman et al. (2008) used data from four waves to identify five PYD developmental trajectories exhibited by adolescents from Grades 5 through 8. Three trajectories remained relatively stable over time, labeled as “high” (27.6%), “medium-high” (39.6%), and “low” (7.8%). The remaining two trajectories showed a slow increase (“increasing,” 21.3%) or decrease (“decreasing,” 3.7%) over time. Similar gender differences were again identified in this study.

With a longer follow-up period, four PYD trajectories were identified among Grades 5–10 students using data from six waves, with the majority of adolescents clustering in the two highest trajectories (Lewin-Bizan et al., 2010). These two trajectories exhibited growth from the initial point to a stable high level after sixth grade (i.e., “increasing-to-stable-high,” 28.3%) and a stable moderate level (i.e., “increasing-to-stable-moderate,” 39%). The other two trajectories included one that grew from the initial point to sixth grade and then began to decline continuously (i.e., “increasing-decreasing,” 26.5%) and another that showed a gradual decline over time (i.e., “decreasing,” 6.2%). Consistent with previous findings (Phelps et al., 2007; Zimmerman et al., 2008), girls are more likely to be in the optimal developmental trajectory, namely, in the “increasing-to-stable-high” group. Using a sample from Grade 7 to Grade 9 students, Schmid et al. (2011) reported four PYD developmental trajectories, with three representing moderate (27%), high (42%), and highest (24%) groups that remained stable over time. A smaller proportion of participants (7%) clustered in the fourth trajectory, which had a lower starting point compared to the other groups and showed a continuous slow decline throughout the study. The superior performance of girls in PYD was still observed, with more girls clustering in the optimal PYD trajectory (i.e., the highest group).

Beyond the 4-H Study, other longitudinal studies have investigated the trajectories of PYD among adolescents in Western cultural contexts. Using four waves of data, Kaniušonytė and Truskauskaitė-Kunevičienė (2021) identified three PYD trajectories among Lithuanian adolescents from Grade 9 to Grade 12, specifically, “high” (30%) and “medium” (48%) remained stable, while “low” (22%) showed a slight decline from Grade 9 to Grade 10, followed by a slow and slight increase. However, no significant gender differences were observed in this study. In another study focusing on Latinx adolescents (Coulter, 2022), using three waves of data, researchers examined adolescents from Grade 8 to Grade 10 and observed that their PYD trajectories experienced a slight decline from Grade 8 to Grade 9, followed by a more pronounced decrease from Grade 9 to Grade 10. Other longitudinal studies can be seen in the handbook by Dimitrova and Wiium (2021) and their review (Wiium and Dimitrova, 2019).

In the Chinese context, drawing upon the theoretical framework of 15 constructs of PYD summarized by Catalano et al. (2004) and using the “Chinese Positive Youth Development Scale (CPYDS),” Shek and Lin (2017) examined the developmental trajectories of PYD attributes among adolescents in Grades 7 through 12 in Hong Kong. The study revealed that the overall developmental trajectory of PYD followed a quadratic pattern rather than a linear one, with a decline observed from Grade 7 but a later increase after Grade 10. Similar developmental trajectories were also found in three higher-order factors: “general positive youth development,” “cognitive-behavioral competence,” and “positive attributes,” based on the four higher-order components identified by Shek and Ma (2010), with the fourth factor being “positive identity.” These findings replicated the results of their previous work (Shek and Ma, 2012), which utilized 8-wave data from Grades 7 to 11 in Hong Kong and observed a U-shaped curve in composite PYD attributes (e.g., moral, behavioral competence and resilience), initially declining, then increasing, and gradually declining again. Moreover, Shek and Zhu (2020) investigated the developmental trajectories of thriving among Hong Kong adolescents. Utilizing 8-wave data from Grades 7 to 11, the overall thriving developmental trajectory also exhibited a U-shaped curve. Specifically, thriving declined from Grade 7 to 9 and then began to rise again from Grade 10 onwards.

In recent years, researchers began some related studies in mainland China. Measured by the CPYDS, Chi et al. (2020) reported a similar change pattern of positive identity among early adolescents from China, showing a decline from Grade 7 to Grade 8 followed by a rebound in Grade 9. However, they observed in the study that the other three components of PYD constructs were relatively stable from the starting point and then slightly increased in Grade 9. Utilizing a similar measurement tool, Chai et al. (2023) explored the trajectory of PYD among high school students during the first semester in China. They identified four trajectory types of dynamic changes in PYD, which were “high start-fast decreasing” (5.0%), “high start-low decreasing” (61.1%), “low start-low increasing” (8.3%), and “mid-persistent” (25.6%) groups. These findings highlighted the dynamics and diversity of adolescent PYD development trajectories, that is, multi-finality of the same initial level and convergence of different initial levels at the endpoint.

Although some preliminary empirical evidence has been obtained using samples of Chinese adolescents, compared to Western contexts, research on the developmental trajectories of PYD among adolescents in Chinese contexts remains limited, highlighting a significant gap in the literature. When using “longitudinal” and “adolescent” as keywords to search in PsycINFO database in November 2024, there were 4,289 articles obtained. By adding “Chinese,” the number dropped to 84. When searching with PsycINFO database utilizing “PYD,” “longitudinal” and “adolescent,” we found 7 articles, and after adding “Chinese,” we did not find any record of publication.

### 1.3 Family functioning as the foundation of positive youth development

PYD is concerned with the dynamic relationship between individuals and their developmental contexts. Within the “relational developmental system,” the family serves as the earliest and most significant microsystem of every child, exerting enduring influences on their development (Xia, 2022). Parents are considered as the primary proximal factors directly impacting children’s developmental outcomes and well-being (Yu and Shek, 2021; Shek et al., 2022b). It is widely acknowledged that healthy family functioning forms the foundation for the positive development of the youth (Smith et al., 1997; Stormshak and Dishion, 2002) which is determined by a variety of interrelated factors (Coulacoglou and Saklofske, 2017). In different models of family functioning, it is commonly regarded as a concept with different dimensions (Beavers and Hampson, 2000; Miller et al., 2000; Olson, 2000), such as problem-solving, communication, and emotional involvement in the family.

Using cross-sectional adolescent samples, healthy family functioning has been demonstrated to be a promotive factor for adolescents’ positive development. For instance, a strong positive association between positive family relationships (e.g., satisfactory relationships with parents, and frequent participation in family activities) and PYD among adolescents was observed in Grasmeijer et al. (2024). Notably, these positive associations were stronger among girls compared to boys. It was reported that healthy family functioning reduced adolescents’ internalizing problems by facilitating PYD (Wang et al., 2021). Leung et al. (2016) observed that in single-mother families, single mothers’ perceptions of family functioning level were positively associated with their adolescent children’s developmental positive outcomes. Moreover, Mackova et al. (2019) examined more complex pathways that connected family crisis (a risk factor for healthy family functioning) with PYD in adolescents: crisis in the family leading to perceived poor parental supervision which reduced participation in family activities, eventually leading to declined PYD.

### 1.4 Family functioning and positive youth developmental outcomes in Chinese families

Chinese society is often regarded as familial, as Chinese individuals prioritize the interests of the family over their own personal interests (Yan, 2018). However, the structure of the family has undergone transformation, transitioning from the traditional three-generation household to the nuclear family in contemporary China. This nuclear family structure was further reduced to couples with only one child after the implementation of the “One-Child Policy” by the Chinese government in 1979. Although this policy officially ceased on January 1, 2016, the nuclear family remains prevalent in modern Chinese society. Consequently, “child-centeredness” in traditional Chinese families has become more pronounced in the present-day Chinese family context (Xu et al., 2007), with children receiving the utmost attention within the Chinese family system. Despite these changes, Confucianism continues to hold a central position as a cultural value influencing family relationships and family education in contemporary China. This traditional philosophical ideology highlights the importance of group harmony over individual fulfillment (Wong, 2002). In light of the traditional values that emphasize harmony and mutual obligations, Chinese families tend to have a low tolerance for open family conflicts and a high emphasis on family cohesion (Chow, 1999). Harmony is considered a prominent facet of family strength in Chinese families, and the provision of family support contributes to enhanced family functioning in China (Xie et al., 1996, 2004).

Research supported the impact of healthy family functioning on developmental problem behaviors in Chinese adolescents. For example, Shek et al. (2022b) found that healthy family functioning is not only a negative predictor of juvenile delinquency but also a positive predictor of PYD attributes among young people from mainland China. Using positive developmental outcomes, Shek et al. (2014) observed significant positive correlations between various aspects of healthy family functioning and PYD in multiple domains across each wave. Dou et al. (2023) also demonstrated that healthy family functioning predicted an increase in resilience in children from mainland China over time.

On the other hand, the highly child-centered nature of modern Chinese families is often coupled with high parental control, including both behavioral and psychological control. Research on Chinese adolescents in Hong Kong indicated that adolescents’ life satisfaction was associated with parental behavioral control, while their sense of hopelessness was related to parental psychological control (Leung and Shek, 2020). Additionally, the self-esteem of Chinese adolescents was positively predicted by parental autonomy-granting behavior (Bush et al., 2002). Nevertheless, it was observed that as children enter preadolescence, American parents are more likely than Chinese parents to reduce their control, such as making fewer decisions about their children’s personal issues. Consequently, children from American families exhibited better emotional functioning (emotional competence) during early adolescence and late adolescence (Qin et al., 2009).

### 1.5 Research gap in the current literature

Upon a closer examination of the existing literature, two significant study limitations are present. First, despite the multiple studies on the developmental trajectories of PYD during adolescence, there is a notable lack of investigation during preadolescence (which is commonly regarded as a period between 9 to 12 years; Buhrmester, 1990; Verburgh et al., 2014), especially in a Chinese context. Preadolescence is considered a unique period during which preadolescents undergo concurrent physical, cognitive, and social–emotional changes, manage changes in school and social relationships, and address increased conflicts in interpersonal relationships, which warrants specific attention (Mascia et al., 2023). Recently, some limited preliminary findings were provided by Chen et al. (2024). They used the three-wave data from Chinese young individuals to estimate the trajectory for PYD attributes during late childhood and adolescence. The developmental trajectory for PYD attributes among the participants was observed as nonlinear, with a first drop and a later increase across the 3 years investigated. However, the participants involved in Chen et al.’s study (2024) had a wide age range, from 7 to 21 years old, which not only implies significant individual differences but also notable disparities in psychosocial competence, thereby affecting the generalizability of the results.

Secondly, there is limited knowledge regarding the longitudinal dynamic relationship between family functioning and PYD among preadolescents and adolescents in the Chinese cultural context (Shek et al., 2022b). With growing urbanization and Westernization, families in China are facing increasing challenges, such as growing divorce and family violence. Besides, with the influence of traditional Chinese cultural influences, Chinese family members may have different views of family functioning, such as the expression of negative views. Hence, it is theoretically and practically important to understand the linkage between family functioning and child developmental outcomes in Chinese societies.

### 1.6 The present study

Based on 15 PYD constructs model by Catalano et al. (2004), the present study investigated PYD trajectories among Chinese preadolescents and adolescents. In response to the research gaps discussed above, this study aimed to answer the following questions.

Research Question 1: What is the development trajectory for PYD attributes in Chinese preadolescents and adolescents? Based on the available findings in samples of Chinese adolescents from Hong Kong (Shek and Ma, 2012; Shek and Lin, 2017), it was predicted that there would be a decline in PYD development with a later rebound during preadolescence and adolescence (Hypothesis 1).

Research Question 2: Is gender related to the initial level and changes in PYD trajectory in Chinese preadolescents and adolescents? Previous studies have shown that girls are more likely to have a more optimal PYD development trajectory compared to their male counterparts (Phelps et al., 2007; Lewin-Bizan et al., 2010). Hence, we expected that compared with boys, girls would be more likely to have a higher level of PYD at baseline (Hypothesis 2a) and have a faster growth rate in PYD (Hypothesis 2b).

Research Question 3: How do the initial levels and the subsequent development of PYD and family functioning co-vary across time among Chinese preadolescents and adolescents? With reference to the promoting effect of healthy family functioning on positive outcomes among adolescents, we proposed that as levels of family functioning increase, PYD would also increase (Hypothesis 3).

## 2 Materials and methods

### 2.1 Participants and procedure

The current longitudinal study conducted four waves of surveys on Chinese preadolescents and adolescents. The initial data collection took place in 2019/20 academic year, from December 2019 to January 2020 (T1), before the outbreak of the pandemic. Using a cluster sampling method, five schools (one elementary school, one junior secondary school, and three admitting elementary and junior secondary students) were selected to participate in this study from among 623 elementary schools, 317 junior secondary schools, and 156 schools that admitted both elementary and junior secondary students located in Chengdu, Sichuan Province, China. A total of 143 classrooms in the five schools involved participated in the study. To specifically assess the impact of the pandemic on Chinese students, a second survey was conducted after classes resumed in the same academic year, specifically from June 2020 to July 2020 (T2). Subsequent surveys were conducted at one-year intervals, with T3 in June 2021 (in 2020/21 academic year) and T4 in June 2022 (in 2021/22 academic year), to more comprehensively track and capture the developmental trajectories and changes in students over time.

This study focused on preadolescents and adolescents (i.e., Grade 4 and above). A total of 6,854 students who were in Grade 4 and above completed the questionnaire at T1. Among them, 748 students participated only in the first survey. One thousand three hundred and fifty-six students participated in any two surveys, with 1,331 completing the survey at T1 and T2, 14 completing at T1 and T3, and 11 completing at T1 and T4. In addition, 2,071 students participated in any three surveys. Among them, 1,759 completed the survey at T1, T2 and T3, and 312 completed the survey at T1, T2, and T4. A total of 2,679 students participated in all four surveys. Hence, the analytical sample for this study was drawn from these 2,679 data points. Inclusion criteria include (1) students who participated in all four waves of the survey; (2) the overall missing rate of less than 50%; (3) less than 10% missing data for the variables of interest. The sample size included in the final analysis was 2,652. The study sample comprised students from Grades 4 through 8 at T1. Specifically, there were 853 students who were in Grade 4, 232 in Grade 5, 742 in Grade 6, 816 in Grade 7, and 9 in Grade 8. According to T1 data, the average age was 10.94 years (SD = 1.32; range 9–15), and 51.1% were male (*n* = 1,354). According to Buhrmester (1990), 89.8 and 10.2% could be regarded as preadolescents (9–12 years old) and adolescents (over 12 years old), respectively, in the first wave of survey.

Students completed the questionnaire in the classroom during each of the four survey rounds. A trained and experienced researcher was present during each data collection session, providing detailed explanations of the research purposes to the students. Scholars and educators hold the view that elementary school students in the upper grades, namely, those in Grade 4 and above, possess adequate cognitive and intellective abilities to comprehend social-psychological factors and engage in self-reporting (He and Xiang, 2022). Nevertheless, the elementary school participants were instructed to ask the researchers any questions they had about the questionnaires before and during its completion, and to respond according to their true feelings and understanding. Both parents and students provided their informed consent before participating in the study. This research obtained ethical approval from the Institutional Review Board (IRB) at Sichuan University.

### 2.2 Measures

#### 2.2.1 Positive youth development

The 90-item “Chinese Positive Youth Development Scale” (CPYDS; Shek et al., 2006) was used to assess PYD attributes. The CPYDS consists of 15 subscales that assess 15 attributes of PYD (Catalano et al., 2004). Each of the first 83 items was rated on a 6-point Likert scale (1 = “strongly disagree,” 6 = “strongly agree”), while the last seven items concerning positive behavioral recognition were rated using a 7-point Likert scale (1 = strongly negative description, 7 = strongly positive description). The total score evaluates the overall characteristics of PYD, with higher scores indicating greater PYD attributes. The CPYDS has demonstrated good test–retest reliability and validity in Chinese adolescent samples (Shek and Ma, 2010). In the current investigation, the CPYDS showed excellent internal consistency (see Table 1).

**Table 1:** The mean value, standard deviation, correlation matrix between study variables, and reliability of scales across 4 time points.

	α	Inter-item correlation	Mean	SD	1	2	3	4	5	6	7	8	9
1. Gender^a^					–								
2. Age			10.94	1.32	0.004	–							
3. PYD (T1)	0.974	0.350	5.16	0.72	0.034	−0.035	–						
4. PYD (T2)	0.980	0.408	5.14	0.78	−0.028	−0.094***	0.551***	–					
5. PYD (T3)	0.984	0.460	5.05	0.83	−0.049*	−0.160***	0.400***	0.478***	–				
6. PYD (T4)	0.981	0.428	5.20	0.72	−0.008	−0.027	0.147***	0.124***	0.182***	–			
7. FFT (T1)	0.935	0.324	4.16	0.72	0.035	−0.015	0.493***	0.347***	0.294***	0.093***	–		
8. FFT (T2)	0.941	0.365	4.17	0.73	0.003	−0.087***	0.371***	0.474***	0.363***	0.072***	0.482***	–	
9. FFT (T3)	0.948	0.388	4.18	0.73	−0.025	−0.109***	0.311***	0.368***	0.483***	0.140***	0.410***	0.455***	–
10. FFT (T4)	0.948	0.390	4.27	0.66	−0.004	−0.040*	0.115***	0.100***	0.142***	0.472***	0.102***	0.120***	0.174***

a 1 = male, 2 = female; *p < 0.05; **p < 0.01; ***p < 0.001.

T1 = Time 1; T2 = Time 2; T3 = Time 3; T4 = Time 4; SD, Standard Deviation; PYD, Positive Youth Development; FFT, Family Functioning.

#### 2.2.2 Family functioning

The 33-item “Chinese Family Assessment Instrument” (C-FAI; Shek, 2002) was used to assess family functioning. The C-FAI assesses family functioning across five dimensions: “communication,” “mutuality,” “harmony and conflict,” “parental concern,” and “parental control,” using a Likert scale ranging from 1 (“most similar”) to 5 (“most dissimilar”). Regarding scoring, all positive statements are encoded in a reverse manner, where a higher score on an item was positively correlated with healthier family functioning, with a higher total score indicating better overall family functioning (Shek, 2002). Past research has reported strong validity and reliability (Shek et al., 2023). The C-FAI demonstrated good reliability in this study as well (see Table 1).

### 2.3 Data analytic plan

To capture the trajectory of PYD over time and investigate the concurrent development of PYD and family functioning across four waves, we employed Latent Growth Curve Modeling (LGCM), an analytical approach for tracking changes in variables longitudinally (Kline, 2016). LGCM enables the delineation of a variable’s trajectory (either growth or decline) and the differentiation among individuals in terms of their developmental patterns. Through LGCMs, we calculated the means and variances of the latent intercepts and slopes to determine any significant individual differences in the initial levels of PYD or in the rate and direction of its development. The quadratic slope represents the rate of change in the linear slope over time (Bollen and Curran, 2006). A larger linear slope value indicates a faster rate of change, while a larger quadratic slope value signifies an accelerating rate of change (i.e., a steeper change).

The maximum likelihood estimation with robust standard errors (MLR) was used for all models in this study. Unconditional linear and quadratic LGCMs were first performed to evaluate the overall growth pattern of PYD without any covariates. Next, we included gender as a time-invariant covariate in a conditional LGCM to determine any significant gender differences in the initial level of PYD and the rate of change in the PYD development. Finally, a parallel processing LGCM was performed to assess the parallel growth of PYD and family functioning after controlling gender. Model fit was assessed using Chi-square tests, Comparative Fit Index (CFI), Tucker-Lewis Index (TLI), Root Mean Square Error of Approximation (RMSEA), and Standardized Root Mean Square Residual (SRMR). A good fit is indicated by CFI and TLI values above 0.95 (acceptable if above 0.90), RMSEA values below 0.06 (acceptable up to 0.08), and SRMR values below 0.08. For model comparison, lower Bayesian Information Criterion (BIC) values are preferred, with the smallest BIC indicating the best fit. A BIC difference (∆BIC) between two models <2 suggests weak evidence in favor of the model with the lower BIC, ∆BIC 2–6 shows positive evidence, ∆BIC 7–10 shows strong evidence, and ∆BIC > 10 shows very strong evidence (Raftery, 1995). The LGCMs were performed using Mplus Version 8.3. Descriptive and correlation analysis of study variables as well as reliability of scales were performed using SPSS 28.0.

## 3 Results

### 3.1 Descriptive statistics

Table 1 provides descriptive statistics and correlation analyses. There was a significant positive correlation between PYD and family functioning level at all waves (rs range 0.072–0.493; *p*s < 0.001). A significant negative correlation was found between age and PYD at T2 (*r* = −0.094, *p* < 0.001) and T3 (*r* = −0.160, *p* < 0.001). A significant negative correlation was found between age and family functioning level at T2 (*r* = −0.087, *p* < 0.001), T3 (*r* = −0.109, *p* < 0.001), and T4 (*r* = −0.040, *p* < 0.05), respectively. Gender was significantly correlated with PYD at T3 (*r* = −0.049, *p* < 0.05).

### 3.2 Developmental trajectory of PYD

**Figure 1:**
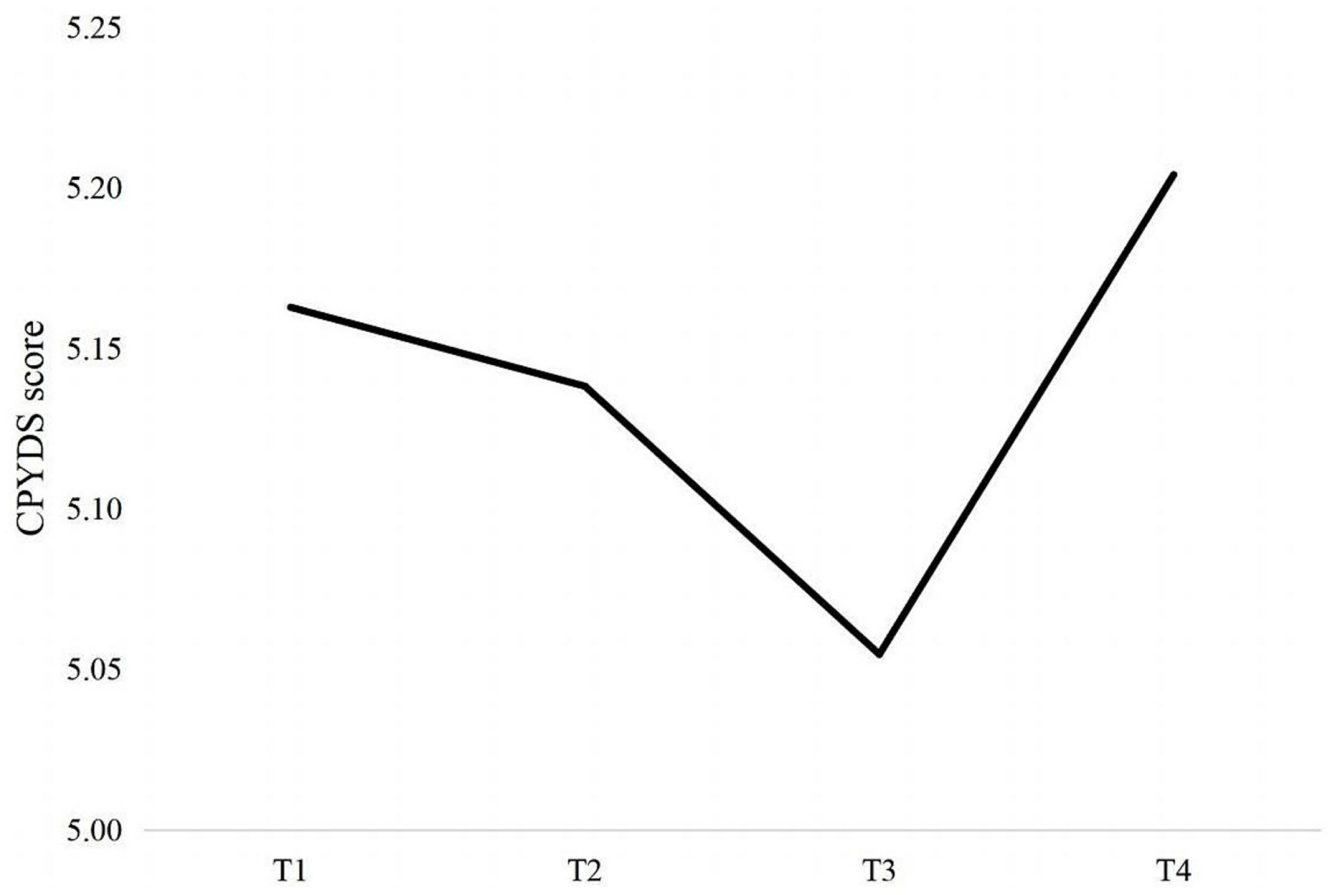
Developmental trajectory of Positive Youth Development (PYD) across 4 waves. T1 =Time 1; T2 = Time 2; T3 = Time 3; T4 = Time 4.

Compared with the linear unconditional LGCM of PYD which reported a relatively poor model fit (see model ft. indicators in Table 2) with a BIC value of 22303.496, the nonlinear (quadratic) unconditional LGCM demonstrated a better fit with a smaller BIC of 22116.651 (∆BIC = 186.85), suggesting a very strong evidence in favor of the model with the lower BIC. Hence, the quadratic unconditional LGCM was used. The parameter estimates are reported in Table 3. Overall, PYD showed a U-shaped developmental trajectory. Figure 1 depicts the average trajectory of PYD over time. Specifically, the means of the intercept of PYD was 5.168, significantly greater than 0 (*p* < 0.001), indicating that students had already developed PYD substantially at the baseline measurement. The means of the linear slope was −0.097 (*p* < 0.001), indicating the instantaneous decreasing trajectory. The means of the quadratic slope was 0.035 (*p* < 0.001), reflecting a U-shape trajectory that decreased firstly and then increased, thus supporting Hypothesis 1.

**Table 2:** Model fit indices of latent growth curve models.

Model	Chi-square	df	CFI	TLI	RMSEA	SRMR	BIC
**Model 1** ^a^							
Linear	250.834	5	0.865	0.838	0.138	0.141	22303.496
Nonlinear	32.550	1	0.983	0.896	0.080	0.020	22116.651
Model 2^b^	32.724	2	0.983	0.916	0.077	0.018	22122.238
Model 3^c^	213.358	12	0.964	0.891	0.080	0.047	41089.325

a Unconditional latent growth curve model (LGCM).

b Conditional nonlinear LGCM with gender as a time-invariant covariate.

c Parallel processing LGCM with gender as covariate.

CFI, Comparative Fit Index; TLI, Tucker-Lewis Index; RMSEA, Root Mean Square Error of Approximation; SRMR, Standardized Root Mean Square Residual; BIC, Bayesian Information Criterion.

**Table 3:** Results of unconditional non-linear LGCM.

	Estimate	*SE*	*p*
S WITH I	0.078	0.073	0.064
Q WITH I	−0.049	0.011	**<0.001**
Q WITH S	−0.059	0.014	**<0.001**
**Means**			
I	5.168	0.014	**<0.001**
S	−0.097	0.018	**<0.001**
Q	0.035	0.006	**<0.001**
**Variances**			
I	0.282	0.037	**<0.001**
S	0.141	0.051	**0.006**
Q	0.027	0.004	**<0.001**

I, Intercept; S, Slope; Q, Quadratic. Bold values indicate statistical significance.

**Figure 2:**
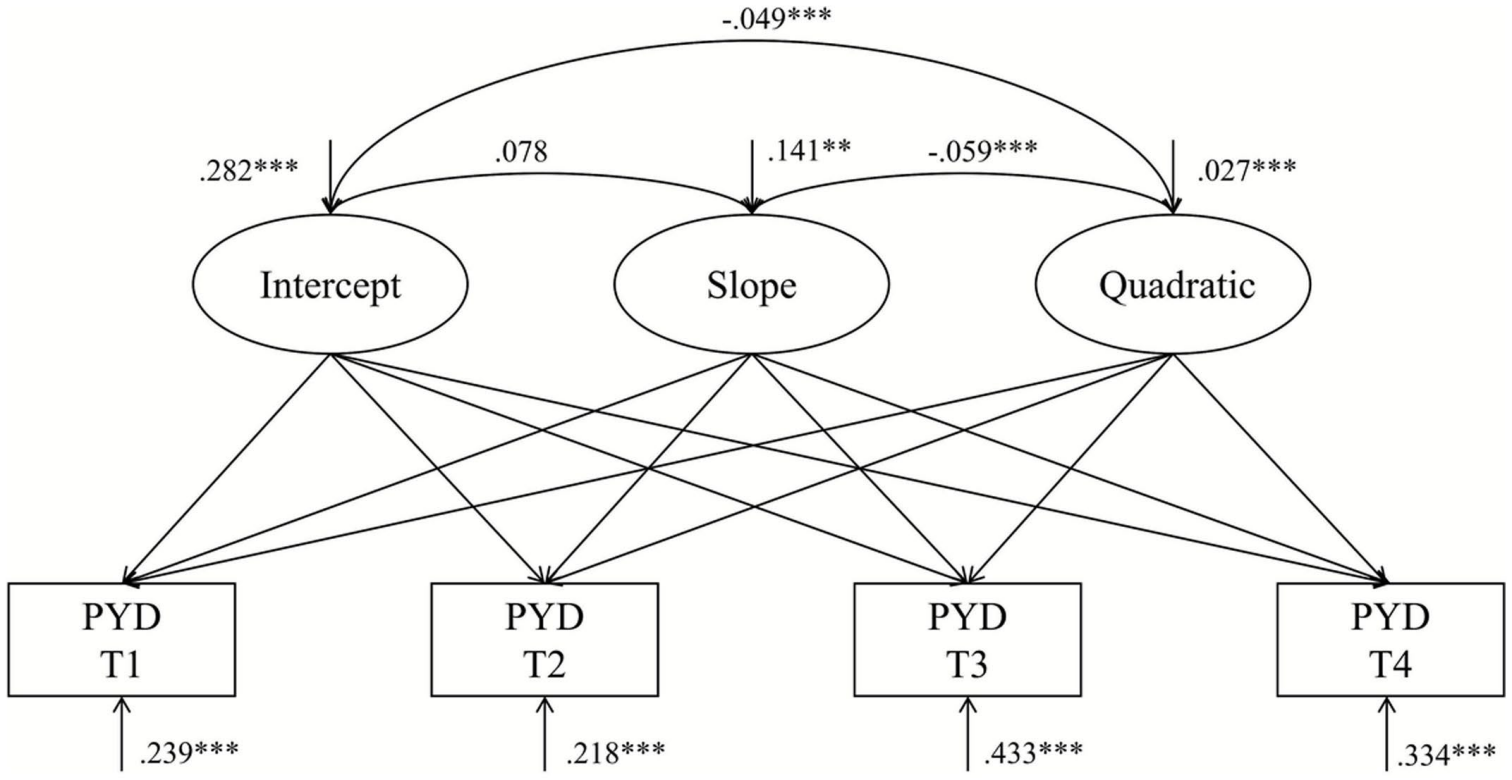
Nonlinear unconditional LGCM of Positive Youth Development (PYD). T1 = Time 1; T2 = Time 2; T3 = Time 3; T4 = Time 4; **p < 0.01; ***p < 0.001.

Moreover, the variance of the intercept (σ^2^ = 0.282, *p* < 0.001), the variance of the slope (σ^2^ = 0.141, *p* = 0.006), and the variance of the quadratic function (σ^2^ = 0.027, *p* < 0.001) were all significantly greater than 0. It suggests significant individual differences in the initial PYD level and the rate of change in PYD. Finally, no significant correlation was found between the intercept and slope (*r* = 0.078, *p* = 0.064). However, the intercept was significantly negatively correlated with the quadratic function (*r* = −0.049, *p* < 0.001). Similarly, the slope was significantly negatively correlated with the quadratic function (*r* = −0.059, *p* < 0.001). The findings indicated that individuals with higher initial PYD levels tend to have decelerated growth over time, as well as individuals with higher linear rates of change tend to have decelerated growth over time (see Figure 2).

### 3.3 Gender difference in PYD trajectory

**Figure 3:**
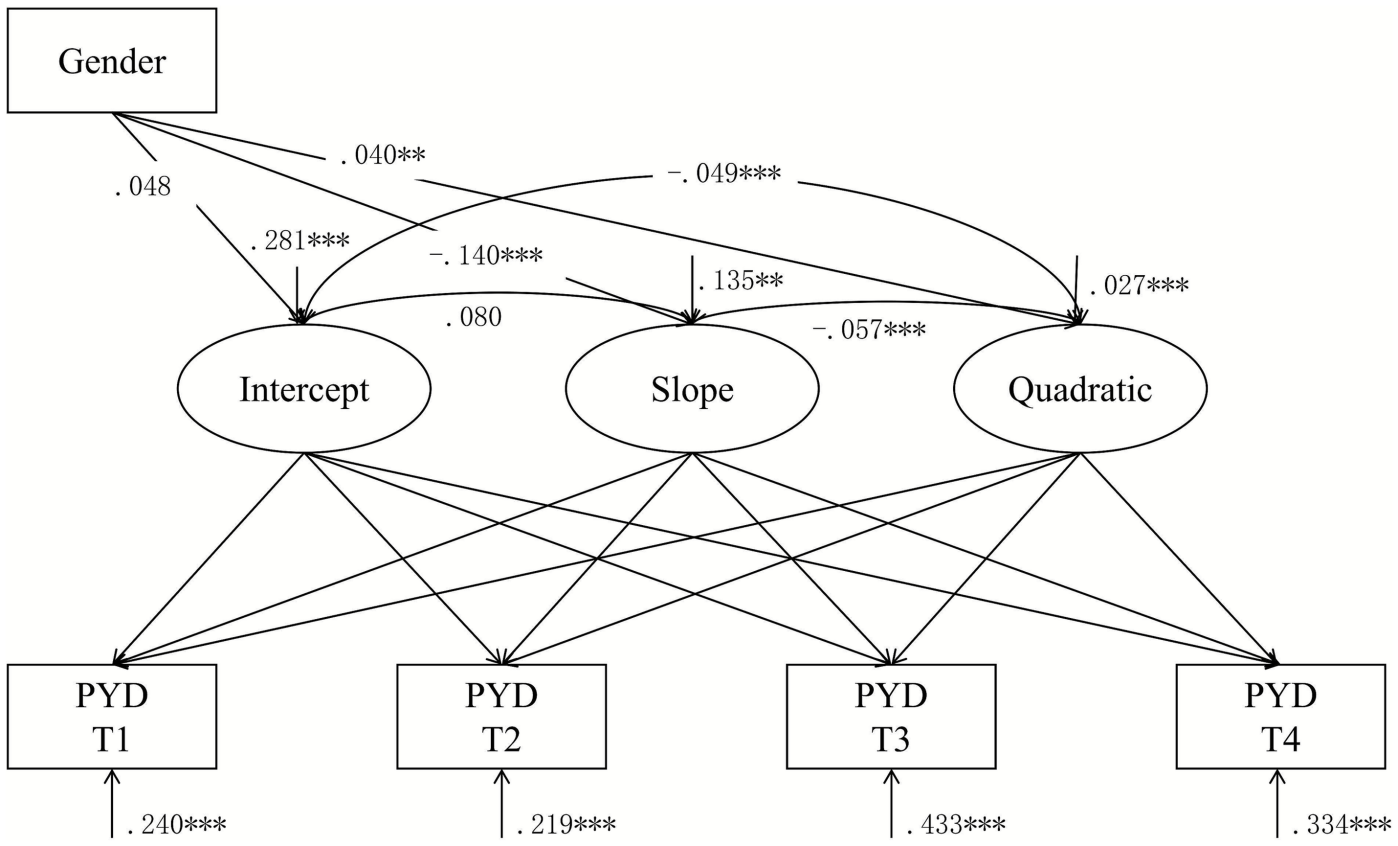
Time-invariant covariate LGCM of Positive Youth Development (PYD). Estimates indicate girls. Gender (0 = male, 1 = female); T1 = Time 1; T2 = Time 2; T3 = Time 3; T4 = Time 4; **p < 0.01; ***p < 0.001.

Conditional nonlinear LGCM, as displayed in Figure 3, adjusted for the time-invariant covariate of gender and demonstrated a good model fit as shown in Table 2 (Model 2). As reported in Table 4, the model results indicate no significant gender difference in the initial PYD levels (β_intercept_ = 0.048, *p* = 0.085). However, the linear slope (β_slope_ = −0.140, *p* < 0.001) and the quadratic slope (β_quadratic_ = 0.040, *p* = 0.001) were significantly influenced by gender. The findings suggested that compared to boys, girls decreased at a slower rate and changed more steeply over time (i.e., accelerated faster). These findings partially supported Hypothesis 2a and Hypothesis 2b.

**Table 4:** Results of conditional non-linear LGCM with gender as a time-invariant covariate.

	Estimate	*SE*	*p*
Gender^a^→I	0.048	0.028	0.085
Gender^a^→S	−0.140	0.036	**<0.001**
Gender^a^→Q	0.040	0.012	**0.001**
S WITH I	0.080	0.043	0.065
Q WITH I	−0.049	0.011	**<0.001**
Q WITH S	−0.057	0.013	**<0.001**

a Gender (0 = male, 1 = female); I, Intercept; S, Slope; Q, Quadratic. Bold values indicate statistical significance.

### 3.4 Joint development of PYD and family functioning

**Figure 4:**
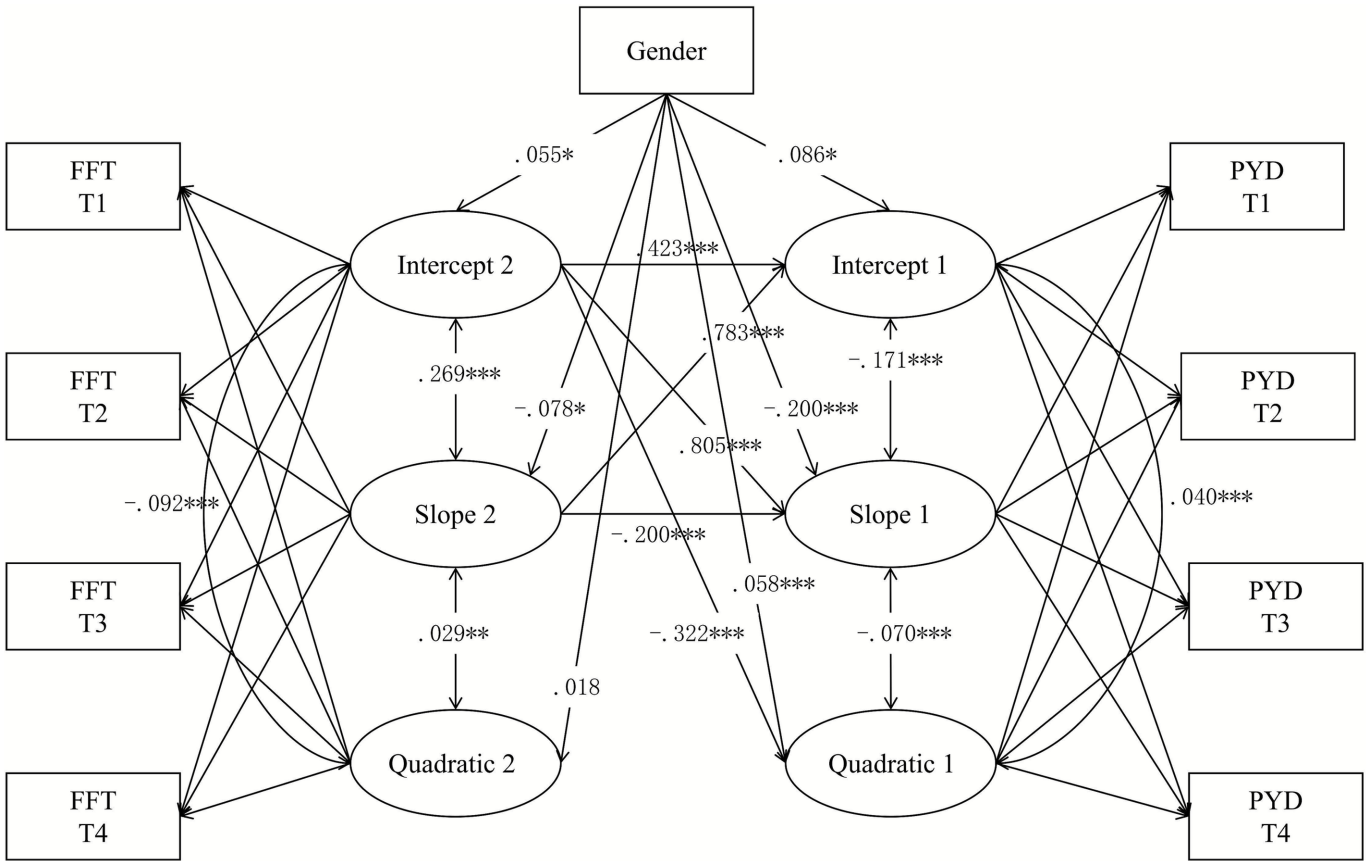
Parallel processing LGCM of Positive Youth Development (PYD) and Family Dysfunctioning (FFT). T1 = Time 1; T2 = Time 2; T3 = Time 3; T4 = Time 4; *p < 0.05; **p < 0.01; ***p < 0.001.

A parallel processing LGCM with gender as a covariate was further tested (see Figure 4), which provided a relatively acceptable model fit (refer to Table 2, Model 3). As presented in Table 5, after accounting for gender, the intercept of family functioning positively predicted the intercept of PYD (β = 0.423, *p* < 0.001), suggesting that better initial family functioning is associated with higher initial PYD levels. Secondly, the intercept of family functioning positively predicted the slope of PYD (β = 0.805, *p* < 0.001), indicating that better initial family functioning leads to a faster positive change in PYD. Thirdly, the slope of family functioning positively predicted the intercept of PYD (β = 0.783, *p* < 0.001), implying that a faster positive change in family functioning corresponds to higher initial PYD levels. Then, the slope of family functioning negatively affected the slope of PYD (β = −0.200, *p* < 0.001), indicating that a faster negative change in family functioning results in a slower rate of change in PYD. Finally, the intercept of family functioning significantly negatively affected the quadratic of PYD (β = −0.322, *p* < 0.001), indicating that worse initial family functioning increases the likelihood of accelerated negative change in PYD. Taken together, Hypothesis 3 was substantiated.

**Table 5:** Results of parallel processing LGCM with gender as covariate.

	Estimate	*SE*	*p*
I2→I1	0.423	0.058	**<0.001**
S2→I1	0.783	0.038	**<0.001**
I2→S1	0.805	0.065	**<0.001**
S2→S1	−0.200	0.016	**<0.001**
I2→Q1	−0.322	0.020	**<0.001**
Gender^a^→I1	0.086	0.034	**0.012**
Gender^a^→S1	−0.200	0.046	**<0.001**
Gender^a^→Q1	0.058	0.014	**<0.001**
Gender^a^→I2	0.055	0.027	**0.040**
Gender^a^→S2	−0.078	0.034	**0.021**
Gender^a^→Q2	0.018	0.011	0.108
I2 WITH S2	0.269	0.014	**<0.001**
I2 WITH Q2	−0.092	0.004	**<0.001**
S2 WITH Q2	0.029	0.009	**0.001**
S1 WITH I1	−0.171	0.039	**<0.001**
Q1 WITH I1	0.040	0.010	**<0.001**
Q1 WITH S1	−0.070	0.013	**<0.001**
Q2 WITH I1	0.003	0.002	0.048
Q2 WITH S1	−0.008	0.002	**<0.001**
Q2 WITH Q1	0.005	0.001	**<0.001**

a Gender (0 = male, 1 = female); 1, Positive Youth Development; 2, Family Functioning; I, Intercept; S, Slope; Q, Quadratic. Bold values indicate statistical significance.

## 4 Discussion

In this study, we examined the trajectory of PYD in preadolescence and adolescence in China and investigated gender differences in the initial levels as well as changes in the developmental trajectory of PYD over time among the participants. Additionally, the study explored the longitudinal parallel change patterns over time between PYD trajectory and family functioning among these Chinese preadolescents and adolescents. Three models were conducted within the LGCM framework to test our three hypotheses. Firstly, utilizing 4-wave data, an unconditional LGCM was employed to assess the univariate growth curve of PYD, revealing a nonlinear U-shaped trajectory with an initial decline followed by an increase. Secondly, a conditional LGCM, adjusting for the time-invariant covariate of gender, was conducted to examine gender differences in initial PYD and the rate of change in the trajectory of PYD over time. The results indicated no significant gender difference in PYD at the baseline. However, girls showed a steeper positive growth in PYD and a slower decline rate in PYD over time as compared with boys. Thirdly, a parallel processing LGCM with gender as a covariate was utilized to evaluate the association between the developmental trajectory of PYD and family functioning over time. Our findings suggested that the initial level of family functioning positively predicted the initial level of PYD and its rate of positive change over time. These findings will be further discussed in detail in the subsequent sections.

Confirming Hypothesis 1 and aligning with prior research conducted on Chinese adolescent populations in Hong Kong (e.g., Shek and Lin, 2017), the overall developmental trajectory of PYD in Chinese preadolescents and adolescents did not exhibit a relatively stable upward or downward trend but rather displayed a fluctuating quadratic U-shaped curve, marked by a decline from T2 to T3 followed by a significant rise. This developmental trajectory suggests that these young individuals may have encountered turbulence in their developmental journey. The study spanned from January 2020 to June 2022, during which students experienced the outbreak of the COVID-19 pandemic, periods of home isolation, interruptions in school activities, and subsequent resumptions. The multifaceted challenges and adaptive demands stemming from this series of significant life events likely contributed to the sustained decline in PYD observed from the initial phase (January 2020) to T3 (June 2021), with a particularly pronounced drop between T2 (June 2020) and T3 (June 2021). Earlier research has documented the enduring impact of the pandemic on young individuals (Shek et al., 2022a), even post-lockdown and resumption of activities (Shek et al., 2021). As noted recently by Shek (2024a), the possibility of “post-pandemic stress disorder” as a consequence of the stressors following the pandemic outbreak is easily overlooked. Such trauma and harm may manifest in various forms, with a decline in the construction and quality of PYD being one manifestation (Wang et al., 2023). The return to school and resumption of regular activities may have introduced additional stressors for children (Rashid et al., 2022; Shek, 2024a), thereby adding extra pressure for these young individuals in this developmental phase with limited coping strategies and socio-psychological resources. As daily life and learning gradually resumed over an extended period, students may have eventually overcome these difficulties and challenges through adaptation and resilience, consistent with the assumption of relative plasticity in human development (Lerner, 1996; Belsky and Pluess, 2013).

Besides the contribution of COVID-19 which is transient in nature, the PYD trajectory may also be an intrinsic developmental change in young people. Across samples of Chinese young individuals from different Chinese communities, such as Hong Kong (Shek and Lin, 2017) and mainland China (Chi et al., 2020), similar nonlinear U-shaped trajectories of PYD suggest that young individuals experience comparable turbulent changes during their development. In contrast, adolescents in Western cultural contexts tend to exhibit relatively stable PYD trajectories (e.g., Lewin-Bizan et al., 2010; Schmid et al., 2011). This difference may be related to the relatively high developmental pressures faced by Chinese children and adolescents. Chinese communities generally place a strong emphasis on academic achievement (Li, 2017), leading to strong expectations for better academic performance at a younger age (Fang et al., 2018). Schools in Chinese communities are also perceived to have stricter discipline and a higher competitive environment (Ang, 2017; Zhou and Kim, 2006). These developmental pressures and challenges may contribute to the initial decline or fluctuation in the PYD trajectories of Chinese young individuals.

Additionally, the results of the quadratic unconditional LGCM also indicated the individual differences in the development of PYD in preadolescents and adolescents. Basically, students exhibited notable variations in their initial levels of PYD and rates of change. Students with higher initial levels and steeper linear growth rates tended to demonstrate a decelerated growth trend in PYD over time. These observations may reflect the possibility of resource constraints that preadolescents and adolescents experience in their PYD development over time. For instance, sustained high levels of functioning may lead to subsequent growth deceleration and resource depletion, as documented in the research literature on human flourishing and well-being (Fredrickson and Losada, 2013). Similarly, developmental theories such as the lifespan perspective (Baltes, 1987) suggest that human growth trajectories typically follow nonlinear patterns, positing that a period of decelerated change often follows a phase of accelerated growth. Consistent with this, prior research has indicated that, overall, PYD development may undergo declines over adolescence (Conway et al., 2015), with different attributes of PYD such as competence, confidence, and connection slowing down or declining over time (Geldhof et al., 2014).

Our findings partially supported our Hypothesis 2, indicating that girls outperformed boys in the development of PYD in adolescent years. Girls demonstrated a slower rate of decrease in PYD and exhibited a steeper positive growth rate of PYD over time, as compared to boys. However, contrary to our prediction, no significant gender difference was found in the initial PYD levels. Previous research suggests that boys and girls may have different developmental advantages in various PYD attributes. For instance, Shek and Wu (2014) observed that girls tend to score higher than boys in terms of pro-social attributes, while boys excel in positive identity and cognitive-behavioral competencies. Female primary school students tend to develop communication skills, empathy, and autonomy experiences earlier than their male counterparts (Sun and Stewart, 2007). On the other hand, male primary school students are more likely to exhibit stronger positive competence beliefs and greater confidence in subjects such as mathematics and physical activities compared to girls (Eccles et al., 1993). In this study, we used the overall PYD score to represent the students’ general PYD characteristics without exploring individual PYD attributes separately. Therefore, it is possible that boys and girls may differ in the initial levels of specific PYD attributes. Another possibility is the “One-Child Policy” in China, which suggests that boys and girls may receive similar family input, hence resulting in similar PYD attributes.

Nonetheless, our findings indicate that girls exhibit a more positive PYD developmental trajectory during preadolescence and adolescence, characterized by a slower decline and a more pronounced positive growth trend. During childhood and adolescence, girls may exhibit greater adaptability and developmental capacity across different dimensions of abilities compared to males (Juraska, 1986). Conversely, boys often exhibit greater vulnerability in their developmental journey compared to girls. They may struggle more to effectively cope with significant stressors during childhood, rendering them more susceptible to developmental challenges, while females appear to demonstrate more resilience in adverse and extreme circumstances. Previous work by Shek and Zhu (2020) provided indirect supporting evidence. Following a longitudinal investigation involving eight waves of data collection on Hong Kong adolescents, they found that interventions aimed at promoting PYD had a more pronounced effect on girls, with the upward trend in PYD manifesting earlier in females compared to their male counterparts. In contrast, boys appeared to require more time to respond to these PYD-promoting interventions and exhibit growth in PYD.

For Hypothesis 3, aligned with our prediction, this study substantiated the reciprocal influence between family functioning and the developmental trajectory of PYD over time, thereby exhibiting a parallel common trend of change longitudinally. After adjustment for gender, it was observed that the initial family functioning positively predicted both the initial level and subsequent rate of change in PYD. Specifically, better initial family functioning was associated with a higher initial PYD level and a faster growth rate in PYD. In other words, entering preadolescence, children in functional, harmonious, and supportive family environments not only commence with higher levels of development at the outset, but they are also more likely to demonstrate faster growth during subsequent development. These observations echo the findings from prior research within the Chinese cultural context where healthy family functioning has been linked to enhanced well-being in children, contributing to positive outcomes such as increased resilience and life satisfaction among young individuals (Dou et al., 2023). According to ecological systems theory, the family environment serves as the most direct and influential external context that shapes children’s overall growth and well-being during childhood and early adolescence (Bronfenbrenner, 2000). Our findings suggest that the family environment, including its static features (harmonious atmosphere) and dynamic features (mutuality and stable family structure), has direct and subsequent developmental implications for PYD during preadolescence and adolescence.

The present findings regarding a parallel change pattern between family functioning and PYD trajectory are noteworthy in a Chinese context. On one hand, the traditional Confucian ideology and the predominant familism within Chinese communities emphasize the importance of family harmony. This value system may to some extent promote changes in parental behaviors and interactions aimed at avoiding family conflicts, thereby fostering a more harmonious family environment for Chinese children. Concurrently, however, the strong emphasis on respect for elders and filial piety within Confucian culture may contribute to authoritarian parenting practices and excessively strict parenting styles among Chinese parents. Based on the observations of Qin et al. (2009), Chinese parents are less likely than parents from Western cultural backgrounds to reduce their control as their children enter preadolescence. In such a family environment with higher levels of parental control, children are less likely to openly express themselves (Dou et al., 2020). Our research findings support the scholars’ viewpoint that children in Chinese families may benefit from reduced parental control and increased autonomy when it is more normative in contemporary families (Qin et al., 2009). Furthermore, child-centered Chinese families commonly prioritize the future success of their children as a central educational goal. As observed in the research by Leung and Shek (2013), expectations regarding children’s future prospects were dominant factors in Chinese parenting styles and family functioning. This lack of supportive family environments, characterized by high levels of parental control and overly stringent parenting styles, led to higher levels of compliance among children from Chinese families, thereby compromising their autonomy acquisition (Huang and Lamb, 2014). This may further impede their development in various areas such as creativity and critical thinking. Autonomy has been identified as a core driving force and key trait for children’s creativity and nonconformity (Kirsch et al., 2015; Wang and Dong, 2019) and is considered one of the fundamental psychological needs of humans according to Self-Determination Theory (SDT; Ryan and Deci, 2000). SDT emphasizes that the fulfillment of children’s autonomy needs is a foundational element for their future positive development and well-being. It asserts that parental autonomy support, specifically addressing the needs for autonomy, plays a pivotal role in promoting children’s intrinsic motivation, adaptability, sense of volition, and broad positive developmental outcomes, including enhanced academic performance, social competence, self-regulation, and psychological well-being (Joussemet et al., 2008; Soenens and Vansteenkiste, 2010).

### 4.1 Theoretical and practical implications

Theoretically, this study supports the thesis that a supportive family environment is a protective factor contributing to positive and beneficial trajectories in PYD during preadolescence and adolescence. At the macro-environmental level, through four longitudinal surveys conducted on Chinese preadolescents and adolescents before, during, and after the pandemic, the study identifies fluctuating PYD developmental trajectories exhibited by students within this turbulent context. Simultaneously, at the micro-environmental level, the research elucidates the dynamic relationship between family functioning and PYD development over time. The contrasting changes in levels of family functioning and PYD trajectory among young individuals underscore the direct impact of supportive family environment on the positive developmental outcomes in young individuals. Overall, these findings collectively indicate that a supportive environment system, encompassing both macro and micro levels, can directly influence the direction and outcomes of young individuals’ subsequent developmental trajectories during preadolescence and adolescence, thereby supporting the notion in PYD models that the occurrence of adaptive individual-environmental developmental patterns serves as a crucial catalyst for positive underage development. Theoretically, there are several insights. First, the study challenges the common belief that adolescents will become fine when they grow, assuming that adolescents will become more mature and stable when they study in high school. Second, the drop in PYD attributes with an increase in age suggests that the higher primary school and early junior years are “high-risk” years. As preadolescence is commonly ignored in the scientific literature (Bulimwengu and Cartmel, 2022), the present study suggests the need to construct more refined theoretical models on late childhood and early adolescence.

Practically speaking, the results of this study highlight the plasticity and resilience of preadolescents and adolescents. The upward trajectory in PYD development over time suggests the potential for growth even in the face of initial maladjustment. Conversely, the decline in PYD development experienced by these young individuals underscores the necessity for additional efforts and timely interventions in nurturing competencies when external environments might pose challenges. Thus, in this regard, the study also emphasizes the benefits of family-based interventions in promoting positive development within the Chinese cultural context. Presently in China, family-centered social service systems such as family counseling remain underdeveloped and not widely accessible. In such circumstances, establishing a network of connections between schools, teachers, and student families is crucial. Moreover, targeted interventions tailored to the unique circumstances of different family structures may characterize this network originating from child-family units. In addition to family services, school-based positive youth development programs that focus on enhancing family bonds can also contribute to strengthening PYD attributes in young individuals (Shek, 2024b; Shek and Dou, 2024).

Furthermore, specific programs or interventions can be designed and developed to ensure synchronized development for children of varying genders. Tailored interventions designed for boys may better address boys’ developmental needs in PYD-related competencies. Additionally, students who initially exhibit higher levels of competence should not be overlooked as being in a “safe zone” of positive development. Ongoing targeted interventions could potentially bridge any developmental slowdown these young people might encounter later on. As Shek et al. (2023) argued, nurturance of individual, family and community resilience is important to help young people adapt to adversities such as the COVID-19 pandemic.

### 4.2 Limitations

Several limitations exist within this study. Firstly, the participants in this research were primarily concentrated in Chengdu City, thus the developmental environments of these students may differ from those of peers in other regions of China, such as rural areas or relatively underdeveloped cities. Consequently, the findings of this study may not be generalizable to a broader young group across different regions in China. Future efforts should aim to capture PYD developmental trajectories in diverse groups of Chinese preadolescents to better comprehend the impact and characteristics of environmental diversity on their PYD constructs and competence development. Moreover, our longitudinal surveys relied on self-reports from students, potentially introducing bias into the results. To more comprehensively capture children’s PYD developmental trajectories and the longitudinal dynamic relationship with family functioning, involving parents and teachers in the assessments would be beneficial. Additionally, utilizing multiple investigative strategies, such as combining self-reports with classroom observations and home visits, could offer a multi-faceted understanding of the dynamic relationship between PYD and family functioning.

## 5 Conclusion

By conducting four waves of surveys on preadolescents and adolescents from China, this study examined the longitudinal developmental trajectories of PYD from preadolescence to adolescence, tested gender differences in these trajectories and the rate of PYD changes, and explored the concurrent changes and relationships between PYD development trajectories and family functioning among these young individuals over time. Utilizing an unconditional LGCM, the PYD developmental trajectories of the participants exhibited a quadratic U-shaped curve characteristic with an initial decline followed by a rebound. According to the time-invariant covariate LGCM, gender differences were observed in change rates of PYD, with girls showing a slower decline rate as well as a faster growth rate in PYD over time. No gender difference was observed in the initial level of PYD. Based on the parallel LGCM, PYD changed in parallel with family functioning. The initial levels of family functioning were positively correlated with the initial level and growth rates of PYD. This study highlights the significance of paying attention to the impact of family functioning in fostering positive development processes among preadolescents and adolescents in the Chinese context.

## Data Availability

The raw data supporting the conclusions of this article will be made available by the authors, without undue reservation.
